# Gunshot Wounds of the Abdomen

**Published:** 1888-03

**Authors:** J. B. Murphy

**Affiliations:** 262 South Halsted Street


### The Medical Journal and Examiner.

VOLUME LVI. MARCH, 1888. NUMBER 3.

### GUNSHOT WOUNDS OF THE ABDOMEN.

BY J. B. MURPHY, M. D.

[Read before the Chicago Pathological Society, Feb. 13.]

CASE I. H. M., age 26, admitted to the Cook County Hospital at 8 p. m., June 16, 1886, suffering from a bullet-wound of abdomen. I saw him at 10 p. m. He was suffering but little from shock; pulse 90; temperature normal; no pain. On examination I found a large bullet-wound half an inch below the umbilicus and an inch to the right of median line. I decided to make laparotomy, and, assisted by Dr. E. W. Lee and the house staff of the hospital, I made the median incision, three and one-half inches long; began examining the small intestine and repairing each wound as it was exposed, by first paring off the ragged edges with a scissors, then inserting a continuous catgut suture in the mucous membrane, then in the peritoneal covering, the first row of sutures being entirely covered by the second. This was continued until the eleven openings were united. I found the bullet had passed through the posterior peritoneal wall close to the pelvis of left kidney. I cleansed the peritoneal cavity with a warm one-half per cent. carbolized water, united the abdominal peritoneum behind with catgut, also the opening made in the anterior peritoneal wall by the bullet; sewed the peritoneum at the incision with a continuous catgut suture; the muscular wall with deep silk suture (interrupted), dressed the parts with iodoform-gauze, then carbolated gauze and borated cotton. The operation lasted two hours; the patient's condition was good after the operation was completed; pulse 110, and of good volume.

The following morning, the house-surgeon reports, "No vomiting, patient slept the greater part of the night, did not complain of pain; pulse 110; temperature $99\frac{1}{2}°$ Fah.; no thirst or tympanitis. At 7 p. m., pulse 96; temperature 99° Fah. No vomiting and no pain; says he is hungry." I called at the hospital the following morning and found the patient suddenly died at 7.30 a. m., after spending a quiet and painless night. At 6 a. m. he complained of weakness and said he was fainting.

*Autopsy.*—Found heart and lungs normal; abdomen filled with blood. The suture in post-peritoneal wall torn through, a large quantity of blood in the retro-peritoneal region, an opening in the left renal artery from which the hæmorrhage came.

The wounds in the intestines were *all completely united, being both air and water tight;* in the majority of these the catgut could not be seen on account of the deposit of reparative material placed about them. There was no evidence of impending gangrene. Though the patient only lived 36 hours after the operation, the peritoneum had already restored itself, and made the openings impervious. There was no evidence of beginning peritonitis. Cause of death, hæmorrhage from renal artery.

CASE II. G. J., colored, aged 22, admitted to the Cook County Hospital May 24, 1887. He said he had been shot in the abdomen about two hours previous.

On examination, a bullet-wound two inches to right of the median line and an inch above the umbilicus was found; there was dullness in the lower portion of the abdomen. Pulse 66, of good volume; there was some shock. I decided on an exploratory incision. I found the abdomen full of blood. The bullet passed from the abdominal wall into the margin of the liver, passing through its substance almost directly backwards and into the muscles of the back. The hole in the posterior surface of the liver could be felt by passing the hand around the liver. There were no perforations of either stomach or bowels. The abdomen was cleansed, about two pints of blood and clots removed; the hæmorrhage had ceased. The opening made by the bullet in the abdominal wall was closed with catgut suture from the inside, abdominal peritoneum united with catgut suture, and the muscular wall and skin with silk. The wound was dressed antiseptically. The operation lasted 30 minutes. The patient was in good condition.

May 25, there was slight tendency to vomit during the night and some pain, but he feels well this morning. The patient's temperature did not reach 99° F. from that time on, nor did he have a single untoward symptom until June 3, when he complained of pain in the back, and on examination a small swelling just over the supposed position of the bullet was found. He was anæsthetized and the bullet removed, also a small quantity of pus. Convalescence was rapid and the patient was discharged June 13.

CASE III. Geo. S., colored, age 57, a musician (but more of a drunkard) by occupation, was admitted to the Cook County Hospital August 23, 1887, at 3 p. m. He had been shot with a 38-calibre bullet a few hours before, his assailant standing fifteen feet in front of him and a little to the right.

The bullet had entered on a level with the 9th rib, 1½ inches in front of the axillary line. His urine contained no blood. Pulse 78, full and strong; no shock; wound had been probed before the patient came to the hospital. Three hours after the injury, the patient was anæsthetized, and an incision was made three inches long downwards and inwards from the point of entrance of the bullet towards the umbilicus. The bullet was found to have passed through the liver ¾ of an inch from its lower margin; also perforating the transverse colon on its convex portion about one-half of an inch from its beginning, leaving a bridge of intestinal tissue half an inch in length between the points of entrance and exit, then passing into the muscles of the back an inch to the right of the spine. The bridge of intestine between the two openings was cut through, and the edges of the wounds freshened, making an opening about one and one-half inches in length. The mucous and muscular layers were first united by a continuous catgut suture; the peritoneal covering was then sewed over it with a continuous catgut suture; about half a drachm of fæcal matter escaped from the intestine after the opening was enlarged, but none before. The abdomen was cleansed with boric acid solution, and blood-clots removed. The parietal peritoneum was united with continuous catgut suture. The muscular and cutaneous tissues were united by interrupted silk suture. The wound was dressed antiseptically. The operation lasted about three-quarters of an hour. The patient left the operating table in good condition; pulse 90, and of good quality; 7.30 p. m., pulse 78; patient vomited considerably in the afternoon, and there was some pain in the wound.

August 24, 8 a. m., pulse 72; temperature 99° Fah.; slept considerable; ordered half-ounce doses of brandy every two hours with carbonated water; 7 p. m., pulse 76, temperature 100.2°; vomited until 4 p. m., but not since; feels hungry and thirsty.

August 25, a. m., pulse 74; temperature 98.5°; slept well; no vomiting. From this time on the patient made a rapid recovery.

The first dressing was removed September 2; there was complete primary union. The sutures were taken out and the wound was redressed. The patient was discharged, cured, September 16. I have seen the patient since then and he says he is feeling as well as he ever did.

CASE IV. J. H., aged 26, was admitted to Cook County Hospital on December 1, 1887, at 8.30 a. m., for gunshot wound of the abdomen.

On examination, a wound was discovered on the right side nearly in the axillary line and just below the costal cartilages, apparently penetrating downwards and inwards. The edges of the orifice were blackened and powder-stained, and another opening on the left side, midway between the costal cartilages and the level of the umbilicus, was found. The patient was suffering profoundly from shock. Under ether, and after the patient had walked two miles, the wound on the right side was enlarged, when it was found that the bullet had entered the peritoneal cavity notching the anterior margin of the liver in its downward course. The usual median incision was made, revealing two wounds in the stomach, with extravasation of a small quantity of food into the abdomen, and one in the mesentery, with some bleeding from the latter. These were all carefully sewed up by deep and superficial catgut sutures. The intestines were drawn out and enveloped in warm towels. No wounds were found in them. The peritoneal cavity was thoroughly washed out with warm solutions of boric acid, and the incisions sewed up with silk after securing the peritoneum with catgut. A rubber drainage-tube was passed through the wound on the right side and brought out on the back above the crest of the ilium. The operation lasted 70 minutes, and the patient was very much collapsed.

Through an error, the patient was given two hypodermic injections of one-third grain of morphia, and he showed severe symptoms of opium-poisoning, and did not become conscious until 6 p. m., some eight hours after the operation. The pulse became rapid and more feeble. At 9 p. m. the patient became delirious and died at 11.30 p. m. There is no doubt that the opium contributed somewhat to the unfavorable outcome in this case, as did also the prolonged operation. The autopsy showed the abdomen clean and no blood in it.

*Remarks.*—There are no positive symptoms of perforation of the stomach or bowels, *i. e.*, if we do not consider the mere fact that a bullet passed through the abdominal cavity *per se* a positive symptom of perforation of the viscera or one of the large vessels. And that we should so consider it is proven by the results of Dr. Parkes' experiments in thirty-eight bullet wounds of the abdomen in dogs, in which only two escaped injury of the stomach and bowels, and in one of those there was considerable hæmorrhage, making only about 2.5 per cent. in which the viscera escaped. Is there any other sign in surgery upon which we act and consider it pathognomonic, where a greater percentage of the cases conform to the law? None that I know of. With this fact staring us in the face, is it not the height of presumption for us to hope that the viscera are not injured after a bullet has traversed the abdomen? Indeed the symptoms of shock were only slight, except in two cases, and in one of them it was due to hæmorrhage.

In case No. 4 there was no vomiting of blood to indicate a wound of the stomach.

At the Ninth International Medical Congress it was suggested by the first speaker on that subject, "that no operation should be performed until such time as fæcal matter appeared at the external wound." This, of course, is absurd, for the food or fæcal material does not escape from the bowel at once after the injury, but it is principally on manipulation that we get an immediate escape, *i. e.*, a primary escape of the contents of the alimentary canal. Nature prevents this escape by an ectropium of the mucous and muscular coats, plugging up the openings temporarily. Dr. Gross states that "extravasation takes place in all cases," but he does not mention the period of time that elapses before it takes place. This is true of the stomach as well as of the bowels, as in case No. 4, not more than a drachm of fluid and food escaped, notwithstanding the fact that the stomach contained a large quantity of food, the two openings would each admit the index finger, and the patient was about for eight hours after the shooting.

In Dr. Parkes' experiments fæcal matter did not appear at the external opening in a single case. This temporary plugging of the opening by the mucous membrane accounts for the tardy appearance of peritonitis in some of the cases of perforating wounds.

When shall we operate and when not?

There are many interesting cases reported of perforating wounds of the abdomen where the patients recover without operation.

Hennen reports a case shot through the abdomen with a ramrod at Badgos, 1812. In the late Rebellion, private Manypenny, as his surgeon records it, "had a ramrod driven plump through his guts." In the Franco-Prussian war, five cases of recovery are reported in one corps, but I cannot find any report of the number of deaths. Larray in a long experience reports only one case of penetrating wound without immediate serious results, and afterwards the bowels were found to be contused. These few remarkable cases are threadbare from being quoted. Abernethy used to shake his head when he had a case of this kind and say, "Nature would have nothing to do with wounds of the small intestines." Bell says, "We announce them as fatal." Sir William McCormac says, in cases of perforation, the median incision should be made as for any other laparotomy and the bowels examined; but, he says, "if a portion of the bowel protrudes from the opening and is not wounded, it should be returned and the opening sewed up." He goes just half way in the right direction and then falters. How can Sir William McCormac tell but that the bowel may be wounded three or six inches from the point of entrance of the bullet? Not any more than he can tell from the examination of the external wound how many perforations there are in the bowels, and that it would be absurd for a man to attempt the latter is thoroughly demonstrated by the excellent and well known experiments of Dr. Charles Theo. Parkes on dogs. He has opened up the field in a most thorough manner and demonstrated to us what can be expected from a bullet passing into the abdomen. *If we will not act it is our fault. Ostrich-like shall we stick our heads in the sand?*

The light which has been thrown on the subject from numerous experiments, and the results following the treatment of cases influenced thereby, puts us in a position where we cannot escape the responsibility from the charge of malpractice when we stand idly by and allow our patient to sink from a septic peritonitis or bleed to death from a wounded vessel. We, as scientific men, should act fearlessly, and, having done so, we will have the consciousness of having fulfilled our whole duty.

All the preparations should be as thoroughly antiseptic as for laparotomies in other cases, *i. e.*, the disinfection of hands, instruments, etc.

The first incision should be to enlarge the opening made by the entrance of the bullet down to the parietal peritoneum, to be sure that the ball entered the abdominal cavity and that it did not rest on the peritoneum as it did in a case of mine. The second incision should be, in the majority of cases, made in the median line, as it allows greater latitude for the examination of the organs; but, in some cases, it will be impossible to bring the wounded bowel to the opening in the median line. When the wound is outside of the mammary line the incision should be over the entrance of the bullet, and enlarged from that point as may be deemed best—that is, in large, broad-bodied men. In all of my experiments on the cadaver I was able to reach the wounded bowel in the most remote parts of the abdomen from the median incision.

The examination of the organs should be methodical and rapid, beginning with the cæcum and examining the small intestines upwards, being careful to keep the bowels as near their normal position as possible, and not to expose them to the air, repairing each opening as it appears. If the exit-opening in the peritoneum can be found, it should be sewed with silk to prevent a return of pus into the abdominal cavity, should any form in the tract or around the bullet. The number of openings in the bowels may vary greatly, and too much care cannot be exercised to make certain that none escape notice and remain open, causing fatal outcome.

Should the mesentery or omentum be wounded, great care must be exercised in ligating the vessels around the opening by suturing the proximal side of the mesentery with catgut, using a full curved, round needle (Czerny), also removing all blood-clots found between both layers of peritoneum in the mesentery.

Another important point is to look for hæmorrhage in the retro-peritoneal cellular tissue, where the ball makes its exit from the peritoneal cavity. Should there be oozing from this opening, it should be enlarged and the vessels closely examined, and, if injured, or oozing, they should be ligated. Had I done this in my first case, I am quite sure the fatal hæmorrhage would have been avoided. I may also mention a case in the hands of one of my colleagues where fatal hæmorrhage occurred from a vein in the retro-peritoneal cellular tissue, in the neighborhood of the left kidney. The patient died of exsanguination on the third day. Autopsy showed the source of the hæmorrhage; also that the patient had had no peritonitis.

The suture to be used is one that (1) can be easily and rapidly inserted; (2) that will keep the serous surfaces in position for forty-eight hours without causing pressure-atrophy; (3) that no suture that is exposed on the peritoneal side of the bowel shall pass through to the mucous surface; (4) that there shall be as little tension on the suture in the peritoneal layer as possible; (5) that they shall be aseptic; (6) that they shall diminish the calibre of the bowel as little as possible; (7) that serosa shall come in contact with serosa.

The materials which at once suggest themselves to fill all the requirements are chromated and juniper catgut, softened in carbolized water, and sublimated silk (1-1000). I used carbolized catgut in my cases, but since that time the reports of Professor Theo. Kocher's experiments and operations in Berne have convinced me that catgut prepared in carbolized oil or alcoholic solutions of the same are not reliable and are frequently septic. The details of the same can be found in the *Correspondenz Blatt für Schweitzer Aerzte*, and are well worth perusal. The best mode of insertion to supply the above-mentioned requirements are (*a*) in longitudinal and small transverse wounds, a double row of continuous sutures, with a round (Czerny) needle, the first approximating the mucosa, and the second the serosa, the latter covering the first from sight; (*b*) in large transverse wounds the Lembert suture should be used. The first can be inserted very rapidly, and the tension on the serosa is lessened by the approximation of the mucosa; it will remain unaltered forty-eight hours and not cause pressure-atrophy, and the serosa unites in from twenty-four to thirty-six hours, as shown by experiments on animals, and in my first case. The continuous suture, exposed on the serous surface, is not exposed on the mucous surface, as in Jobert's suture, but it would diminish the calibre of the bowel in transverse wounds, and for that reason, and that only, is Lembert's suture used in large transverse wounds.

A portion of the bowel must be excised when more than three-fifths of its calibre is destroyed, otherwise there will be a stricture at that point. This can readily be accomplished by invagination with a rubber supporter in the intussusceptum, as suggested by Dr. N. Senn, and a continuous suture approximating the serosa in its entire circumference. This may be further supported by the plastic operation on the omentum to support the bowel in this position and insure adhesions (Dr. Senn). When there are two large openings which can be approximated and leave a fistula, allowing the contents of the bowel to pass through the fistula and the loop of bowel to be retired (if the loop is short, that is, less than four feet in animals), the openings should be approximated by Dr. Senn's bone-tablets, or by the suturing process suggested by one of the surgeons in the late Rebellion. Dr. Reybard's wood-plates may be used and the opening approximated to the abdominal parietes. The danger when the bone and wood plates are used is that they cause pressure-atrophy. These advantages are (1) the rapidity with which they can be inserted, Dr. Senn claiming that he can close the opening in ten minutes; (2) the certainty with which they approximate the serous surfaces and retain them in their position.

*Toilet.*—The blood-clots and escaped contents of the viscera should be removed with a sponge; the abdomen should be washed out with a solution of boric acid until such time as it returns clean from the external wound; the bowel should be replaced as near the normal position as possible, and the omentum spread over the bowel. If it be feared that there is still remaining foreign matter, it would be better to put in a Mikulicz drainage-tube than to lose much time in trying to cleanse the abdomen and thus producing a great degree of shock from which the patient could not rally. *Above all things, profound shock should be avoided.*

262 South Halsted Street.

